# Dysmenorrhea, Endometriosis and Chronic Pelvic Pain in Adolescents

**DOI:** 10.4274/jcrpe.galenos.2019.2019.S0217

**Published:** 2020-02-06

**Authors:** Aalia Sachedin, Nicole Todd

**Affiliations:** 1 The Royal Children’s Hospital, Department of Paediatric & Adolescent Gynaecology, Melbourne, Australia; 2 University of British Columbia, Vancouver General Hospital, Diamond Health Centre, Vancouver, Canada

**Keywords:** Dysmenorrhea, pelvic pain, endometriosis

## Abstract

Most adolescents will experience discomfort during menstruation. Due to normalization of dysmenorrhea, there is delay to diagnosis and treatment. Non-steroidal anti-inflammatories are a first line treatment. Adolescents can safely be offered menstrual suppression with combined hormonal contraception, and progestin-only options. When the above are ineffective, gonadotropin releasing hormone agonists with add back treatment can be considered. Transabdominal ultrasound is indicated when first line treatments do not improve symptoms. Endometriosis should be considered in adolescents who experience ongoing pain despite medical treatment. If laparoscopy is performed and endometriosis visualized, it should be treated with either excision or ablation. Women with endometriosis should be counselled on menstrual suppression until fertility is desired. Management of chronic pain requires the involvement of a multi-disciplinary team.

## Introduction

 The majority (70-93%) of adolescents have discomfort associated with menstruation (1,2). Dysmenorrhea is the most common reason for missed school and activities (3). Up to 20-40% report missed school due to dysmenorrhea, and 40% report a negative effect on school performance and concentration (4). Adolescents with severe dysmenorrhea have impaired quality of life and are at increased risk for depression and anxiety (5). They present later for assessment, see multiple physicians, and suffer more, compared to adults (4). Functional impairment is the primary reason for seeking medical care in adolescents with dysmenorrhea (6). Health care practitioners (HCP) should not normalize dysmenorrhea. Adolescent women should be offered treatment and further investigation for ongoing pain (7).

*Primary dysmenorrhea* is menstrual pain in the absence of pelvic pathology.

*Secondary dysmenorrhea* is menstrual pain in the presence of pelvic pathology or due to a recognized medical condition.

## Differential Diagnosis

Differential diagnosis for pelvic pain is seen in Table 1.

## History

Information about age of menarche, cycle regularity, duration of menses, amount of bleeding, and time elapsed between onset of menarche and dysmenorrhea should be elicited. Pain history should encompass onset, duration, severity, aggravating and alleviating factors, and relationship to menses. Urinary, gastrointestinal, musculoskeletal and psychological symptoms should be documented. The degree of functional impairment, including absence/avoidance of school, social and sports activities should be explored (13). Adolescents should be interviewed with caregivers and independently; parental modeling may influence pain reporting and perception (14). Sexual history, if appropriate, should include presence of dyspareunia, history of sexually transmitted or pelvic infections, and sexual violence. Previous treatment, including medications, dosage and timing should be documented. One study reported that although 70% of adolescents took over-the-counter medications, only 31% followed recommended dosing, and only 16% took analgesics prophylactically (2).

## Physical Examination

Height, weight, and vital signs should be recorded. Further examination can be delayed until a later visit. Prior to initiating hormonal treatment, only blood pressure must be documented (15). HCP should explain the steps and elicit frequent feedback.

**Abdomen:** Light and deep palpation is performed for masses and tenderness. A cotton swab can be used to assess for allodynia (16). Myofascial pain is assessed by asking the patient to contract their abdominal wall muscles while palpating an area of tenderness (Carnett’s test). Increased pain indicates a myofascial trigger point as the intraperitoneal organs are protected (10).

**Pelvis:** The adolescent should be offered a mirror to assist with the educational gynecologic exam. Begin with examination of the external genitalia. Gentle traction of the labia can allow visualization of the introitus. A cotton swab can be used to gently apply pressure to the vaginal introitus to map out points of increased pain, indicating provoked vestibulodynia (17). Internal pelvic examination should be limited to sexually active patients, and may not be feasible due to anxiety, patient expectations, or pain (10). In adolescents that can tolerate a single digit vaginal exam, the bladder neck, levator ani, cervix, adnexa and uterosacral ligaments should be palpated for tenderness. Uterosacral nodules indicative of deep invasive endometriosis are rare adolescents (6,18). If there is concern about obstruction, a moistened cotton swab can be gently inserted into the vagina (19). Digital rectal examination may also be helpful to appreciate hematocolpos; a bulge may be palpated in the vagina.

## Primary Dysmenorrhea

Primary Dysmenorrhea is associated with anovulatory cycles and usually presents 6-12 months post-menarche (8). Pain is due to increased uterine contractility and elevated prostaglandin levels as the ischemic endometrial lining is sloughed off (1,9,20).

**Prevalence:** Dysmenorrhea is estimated to affect 70-93% of young women (1,2).

**Pathogenesis:** Menstrual pain results from vasoconstriction and inflammation. Prostaglandins, leukotrienes and vasopressin are key drivers (21). Omega-6 Fatty acids, including arachidonic acid, are released with progesterone withdrawal, increasing local prostaglandin and leukotriene production (3,21,22). Prostaglandins induce myometrial contractility, vasoconstriction and uterine ischemia (3,21,22). Additional prostaglandin effects include headaches, nausea, bloating, vomiting and diarrhea (21).

**Symptoms:** Discomfort may begin the day preceding and continue during the initial 24-48 hours of menses (4). Nausea, vomiting, diarrhea, headaches and muscle cramps may be present. Pain can be cyclic, acyclic, and/or accompanied by urinary or bowel symptoms (20).

**Investigations:** Primary dysmenorrhea does not require further investigation. Routine ultrasound is not recommended unless first-line treatments fail (11,23).

**Treatment:** Adolescents should be counselled about the menstrual cycle and pathophysiology of dysmenorrhea. Further investigations and pelvic examination are not needed prior to hormonal treatment (11,19). Smoking cessation is recommended, as exposure to tobacco smoke worsens dysmenorrhea (11). Use of a menstrual calendar (paper, e-health app) that is inclusive of pain symptoms, associated symptoms and missed activities may be helpful.

### Analgesia

Non-steroidal anti-inflammatories (NSAIDs) are the preferred first line analgesics; regular use has shown a 27-35% improvement in dysmenorrhea (19,24). No specific NSAID is superior (11,21,25). Adolescents should be counselled to start with twice the regular dose followed by regular dosing (21). If menses can be predicted, NSAIDS should be started 1-2 days prior (26). It is helpful to provide written instructions; nearly 70% of adolescents consumed less than 50% of recommended daily dosing of analgesia (4). Women who experience significant neurological or gastrointestinal side effects should be offered selective COX-2 inhibitors.

### Hormonal

Combined hormonal contraception (CHC) which may be oral, transdermal, or intravaginal, can be offered to adolescents who fail NSAIDs and/or require contraception (21,24). CHC may also be used as a first line option (23). CHC improve dysmenorrhea by reducing endometrial growth, menstrual fluid volume, and prostaglandin and leukotriene production through inhibition of ovulation and decidualization of the endometrial lining (3,21,27). CHC improve dysmenorrhea and reduce missed activities, and can safely be taken cyclically or continuously (10,28). Multiple studies have demonstrated improvement in dysmenorrhea with extended or continuous compared to cyclic regimens (11,15). Progestin-only options may be offered if contraindications to CHC are present. Levonorgestrel-releasing intrauterine systems (LNG-IUS) have been shown to improve both primary and secondary dysmenorrhea (11,29). LNG-IUS are safe to use in adolescent and nulliparous women (30). Please see below for further discussion of hormonal options.

### Complementary

Non-medical interventions including heat, traditional Chinese medicine, acupuncture/acupressure, transcutaneous electrical nerve stimulation (TENS), yoga, and exercise should be discussed (11,19,21). Regular exercise is recommended for all patients with dysmenorrhea (11). Two randomized studies have demonstrated that heat is comparable to ibuprophen (11). Ginger, taken during the first 3-4 days of menses, was superior to placebo and comparable to NSAIDs (11). A recent review demonstrated limited effectiveness for fenugreek, fish oil, fish oil and vitamin B1, ginger, valerian, vitamin B1, sataria, and zinc sulfate (31). Small studies indicate benefit to Omega-3 fatty acid supplementation, high-dose vitamin D supplementation, and low-fat vegetarian diet (21,32). Further research is needed to support acupuncture, acupressure, and TENS (11,33). Patients using complementary medicine should be encouraged to share this with the HCP to reduce medication interactions.

## Secondary Dysmenorrhea

Secondary dysmenorrhea typically appears 12 months post menarche and is associated with progressively worsening pain, chronic pelvic pain (CPP), midcycle or acyclic pain, and irregular or heavy menstrual bleeding (3,8,21). Common etiologies include: endometriosis, adenomyosis and obstructive anomalies.

## Endometriosis

Endometriosis is the presence of endometrial glands and stroma outside of the uterine cavity. Prevalence in adolescents is unknown but has been estimated at 6-10% in reproductive-aged women (34). Endometrial deposits are often seen in the pelvis, but may be present in distant locations such as the upper abdomen (35). In adolescents, peritoneal and ovarian surface endometriosis is most common (36). HCP should have a high suspicion for endometriosis in adolescents with ongoing pain. Younger age has been associated with delay in diagnosis, however more recent studies suggest this is no longer the case (6,35). Approximately two-thirds of women with endometriosis experience symptoms before the age of 20 (37). Even when treated, adolescents with endometriosis experience reduced social and physical functioning compared to their peers (38). The impact on physical and mental health is greater than for young patients with other chronic illnesses (38). Estimated yearly costs for adult endometriosis in Europe, US and Canada ranges between 4000 and 12,000 USD (39).

**Incidence:** Endometriosis is the most common cause of secondary dysmenorrhea. It has been identified in 62-75% of adolescents undergoing laparoscopy for CPP and/or dysmenorrhea and in 70% of adolescents with pelvic pain that did not improve with NSAIDs and/or combined oral contraceptive (COC) (1,40).

**Genetics:** There is a polygenic multifactorial inheritance pattern to endometriosis (24). Twin studies have demonstrated heritability of 51-75% (1,21). Young women with a first-degree affected relative have a 7-10 fold increased risk (35). Epigenetic changes may also play a role (24).

**Risk factors:** These include menarche <14 yrs, shorter cycles, heavy menstrual bleeding, longer duration of menses, obesity, and early onset of dysmenorrhea (8,19,35,41). Obstructive Müllerian anomalies increase risk of endometriosis due to retrograde menstruation (8,24). However, there is often complete resolution of the endometrial implants post restorative surgery (24). Parity and breastfeeding reduce risk (35).

## Implant Pathogenesis

The specific etiology of endometriosis is unclear. Six theories are described:

**Retrograde menstruation:** Sampson’s Theory of Retrograde menstruation is the most widely accepted. Menstrual fluid leaves the uterus via the fallopian tubes and carries endometrial mesenchymal stem cells, epithelial progenitor cells and stromal fibroblasts which attach to the peritoneum (42). Supportive evidence includes endometrial implants found on dependent portions of the pelvis and increased incidence in young women with obstructive Müllerian anomalies (24,39,43). However, it does not fully explain the finding that most women experience retrograde menstruation, yet only 5-10% of adult women have endometriosis (9).

**Coelomic metaplasia:** Peritoneal coelomic mesothelial cells undergo metaplasia and transform into endometrial cells (9,39). This may explain the presence of ovarian endometriosis (39).

**Lymphatic spread:** Endometrial cells travel via lymphatic channels to implant in distant sites (43).

**Hematological spread:** Endometrial cells travel via the vascular system to implant in distant sites (43).

**Immunologic:** Endometrial tissue is able to proliferate due to a decreased cellular immunity (43) and/or women with endometriosis have increased levels of cytokines and growth factors (24).

**Neonatal uterine bleeding:** In this recent hypothesis, neonatal vaginal bleeding is thought to increase the risk of early-onset endometriosis. While actual bleeding is observed in 5% of newborn girls, occult bleeding may occur in 25% (42). At the time of increasing estrogen production (puberty), these endometrial cell clusters are re-activated (36).

## Pain Pathophysiology

Endometriosis is a hormone mediated, neuro-vascular condition (22). The presence of endometrial tissue incites an estrogen-dependent chronic inflammatory reaction (9,20,21). Pain derives from increased prostaglandins, compression and/or infiltration of adjacent nerves (3,21,22,39). Increased expression of nerve growth factor, increased density of nerve fibers, angiogenesis and changes to innervation of the uterus may also contribute (22,35).

## Clinical Presentation

Adolescents may not present with “classical” symptoms: dysmenorrhea, dyspareunia, dyschezia, endometriomas, and/or infertility. Common symptoms in young women with endometriosis include general pelvic pain, low energy and abdominal discomfort (37). Heavy menstrual bleeding, headaches, dizziness, low back pain are also more prevalent (37). Abdominal symptoms can include bloating, constipation, diarrhea, nausea, pain with defecation and pain that improves after bowel movements (6). Severe dysmenorrhea associated with missed activities should raise the suspicion of endometriosis. Onset of menstrual bleeding is not necessary for diagnosis of endometriosis; case reports have described the presence of endometriosis in premenarchal girls with pelvic pain (20).

Symptoms in adolescents with endometriosis are seen in Table 2.

## Investigations

Laboratory investigations can assist with diagnosis (15,19). Initial blood work-up including complete blood count and erythrocyte sedimentation rate may indicate acute and chronic inflammation. Urinalysis and urine culture can identify urinary tract infection and renal/bladder calculus. Pregnancy test and sexually transmitted infection screen should be completed in sexually active patients. There is currently insufficient evidence to support biomarker testing (48).

## Imaging

**Ultrasound:** Given the low probability of endometriomas in the adolescent population, there is debate on requirement for pre-operative ultrasound (7,19,43). Further, a normal ultrasound does not exclude endometriosis, as superficial endometriosis may not be visualized. Müllerian anomalies and other adnexal masses can also be visualised (12). Transabdominal rather than transvaginal ultrasound should be ordered in non-sexually active adolescents.

**Computed tomography scanning:** May assist in the identification of appendicitis.

**Magnetic resonance (MR):** When comparing laparoscopy to MR for the detection of peritoneal endometriosis, MR lacked the likelihood ratio to be routinely used (20). Given most adolescents will be diagnosed with early disease, findings are unlikely to be apparent on MR (7). MR is not cost-effective for investigation of endometriosis.

## Surgery

The gold standard for diagnosing endometriosis is tissue sample. Histological samples of suspicious lesions should be assessed, as there is a high false positive rate for visual identification only (20). Laparoscopy for diagnosis only, without a trial of medical treatments, should be avoided (20). If laparoscopy is undertaken, concurrent treatment of endometriosis should be performed (11,24). HCP may consider speculum examination, bimanual, and pelvirectal exams under anaesthesia based on patient history.

## Management

Endometriosis is a chronic disease. As in primary dysmenorrhea, first-line treatment includes analgesia and hormonal therapy. Endometriosis is an estrogen-dependent disease; most therapies are aimed at supressing ovarian function (20). Most hormonal options are equivalent at reducing pelvic pain; factors such as cost and contraception should be considered in decision-making (20,22,39). CHC or progestin-only options can be offered with anticipated improvement in two thirds of women (24,39). The adolescent should keep a pain journal that logs pain, response to treatment, and other symptoms (19).

**Non-steroidal anti-inflammatories:** NSAIDS can be used prior to expected onset of menses. In endometriosis, there is no clear evidence of a benefit for relief of symptoms compared to placebo (49).

**CHC:** CHC are an ideal first choice due to documented safety, efficacy, low side effect profile and low cost (39). Adolescents with suspected or confirmed endometriosis should be counselled on menstrual suppression to prevent further endometrial proliferation (19,21,22,50). Endometrioma formation and recurrence are reduced through CHC-associated anovulation (50,51). No COC preparation has demonstrated superiority (50). Adolescents who experience ongoing dysmenorrhea with cyclic use can be transitioned to extended cycle. An randomized controlled trial (RCT) demonstrated reduced post-operative dysmenorrhea recurrence in cyclic versus non-cyclic users (52). Another RCT demonstrated improvement in pain scores for both cyclic and extended use, however discontinuation rates were higher in the continuous use group due to unspecified side-effects (53).

**Progestins:** These include oral, intramuscular and LNG-IUS. Progestins demonstrate 80-100% improvement in symptoms due to anti-angiogenic, immunomodulatory and anti-inflammatory effects (22). Side effects include unscheduled bleeding, bloating, breast tenderness, weight gain, and mood changes (24,54). Depot medroxyprogesterone acetate (DMPA) and LNG-IUS are more effective at achieving menstrual suppression compared to oral regimens (28).

Oral progestins can be used to achieve menstrual suppression (Table 3) (28). Medication should be started at lowest dose and increased until menstrual suppression is achieved. Dosage adjustment and compliance is required (28). Progestin-related side effects may be more common in oral regimens (28,54). Norethindrone acetate (NETA) has demonstrated effectiveness for menstrual suppression in adolescents (55). In a Cochrane review, medroxyprogesterone was superior to danazol and equivalent to gonadotropin releasing agonists (GnRHa) at 12 months (54). Dieonogest has selective 19-nortestosterone and progesterone activity (9,56). In the adult population, it has been shown to be equivalent to GnRHa in reduction of dysmenorrhea, dyspareunia, physical symptoms and signs of endometriosis and improvement in daily activities (57). Adolescents requiring contraception should be prescribed oral progestins that are indicated for contraception.

Dosage regimens for progestin only options are seen in Table 3.

LNG is a 19-nortestosterone progestin with anti-estrogen effects on the endometrial lining, thereby inducing endometrial decidualization with resulting endometrial atrophy (58). Ovulation may not always be suppressed. When comparing LNG-IUS and GnRHa (leuprolide acetate), both demonstrate improvement in pain (20,58). Reduced recurrence of pain post-surgery is seen with LNG-IUS (19,58). It is safe to use in adolescent and nulliparous women with 96% success at insertion (30). Higher-dose LNG-IUS is associated with more effective menstrual suppression (28). Adolescents should be counselled on pain with insertion, and cramping/unscheduled bleeding that improves by three months (30).

DMPA can be used safely in adolescents. Users experience improvement in endometriosis and CPP symptoms (56). DMPA suppresses ovulation and leads to amenorrhea by inducing endometrial atrophy. Amenorrhea rates are 55% at one year, and 68% at two years (11). Unscheduled bleeding and weight gain are the most common reasons for discontinuation (59). Use beyond two years is associated with reversible decrease in bone mineral density (BMD) (8,59). At two years post-discontinuation, the BMD was similar to non-users. Further, there is no evidence to support increased risk of fractures and/or osteoporosis (59). Well-counselled adolescents may choose the benefit of symptom control over risk (60). Women using DMPA should be recommended calcium and vitamin D supplementation.

Etonogestrel, an active metabolite of desogestrel, is available as a subdermal implant. Its primary mechanism is anovulation. Improved dysmenorrhea has been reported (59). It is safe for use in adolescents. However, discontinuation may occur due to increase in unscheduled bleeding (30). Counselling young women on common side effects prior to insertion may improve retention rates.

**Anti-progestogens:** Gestrinone, an anti-progestogen, inhibits production and use of progesterone (54). When compared to GnRHa, gestrinone was not as effective at six months, but more effective at 12 months (54). Small studies indicate improvement in pain. Side effects include unscheduled bleeding, acne, weight gain, and fluid retention (54).

**GnRHa:** GnRHa improve endometriosis-related pain by inducing a hypogonadic-state via suppression of the hypothalamic-pituitary-ovarian axis (61). Approximately 90% of users are amenorrheic (43). GnRHa may also improve pain by reducing inflammation, angiogenesis, and inducing apoptosis in endometrial cells (61). In adults, pain can be reduced by 80%, similar to DMPA and LNG-IUD (61). Side effects include hot flushes, vaginal dryness, sleep disturbance, headaches, mood changes and bone loss (21,61). Studies in the adult population suggest addition of letrozole or tamoxifen may reduce these symptoms. GnRHa use in adolescents should be considered second line, after inadequate response to hormonal treatment. For empiric treatment of pelvic pain, initiation of GnRHa should be delayed to 18 years (9). With surgically confirmed endometriosis, the initiation of GnRHa should be delayed until 16 yrs to ensure the majority of bone accrual has occurred (7,9). Intramuscular, intranasal and subcutaneous forms are available, with equivalent treatment outcomes (20). A “GnRHa flare” can occur due to an initial surge of LH and FSH, resulting in increased pain and unscheduled bleeding. To prevent flare, the initial dose should be timed with the late luteal phase (61). Flare symptoms can also be avoided by allowing three-weeks crossover when transitioning from CHC to GnRHa.

Spine BMD can be reduced by 5-8% after 3-6 months of GnRHa use; BMD may not return to baseline once treatment is complete (61,62,63). Based on the threshold theory, “add-back treatment” allows for low estrogen levels to protect bone and reduce vasomotor symptoms without activating endometriotic tissue (20). An RCT demonstrated stability in BMD at 12 months of add-back (64). Adolescence is a time of bone accrual and add-back treatment should be initiated simultaneously with GnRHa (20). This differs from the adult population, whereby add-back treatment is offered after six months. Add-back treatment does not reduce effectiveness (20,24,62). Options include conjugated equine estrogen 0.625 mg with NETA 5 mg, or NETA 5 mg alone (38,61). NETA is converted to ethinyl estradiol and was approved by the Food and Drug Administration for add-back treatment in adults (35,64). Both options have demonstrated improvement in adolescent quality of life, including improved pain, physical symptoms, and social functioning (38).

There is limited research on prolonged use of GnRHa in the adolescent population, and no current guidelines for BMD monitoring (57). Baseline BMD is not required unless there are additional risk factors for osteoporosis (61). Expert opinion suggests that for young women without additional risk factors, a dual-energy X-ray absorptiometry of hip and lumbar spine should be completed after nine months of treatment (7,8,61). BMD should be monitored every two years with ongoing treatment (8). Adolescents should be counselled on calcium and vitamin D supplementation.

**Androgens:** Androgens, such as Danazol, have been previously described to improve dysmenorrhea, but are often avoided due to androgen-related side effects including acne, hirsutism, weight gain, edema, muscle cramping, and worsening lipid profile (20,21,39). Danazol induces endometrial atrophy and has immunosuppressive effects (60). The European Society of Human Reproduction and Endocrinology (ESHRE) advises against the use of Danazol for treatment of endometriosis in adult women (20).

**Anti-androgens:** Cyproterone acetate (CPA) has anti-androgenic and anti-gonadotropic effects. It is available with estrogen in a CHC. CPA has been compared to COC, and both demonstrate improvement in pain at six months (50,56). As CPA can be associated with liver toxicity; liver function should be monitored (54). This may be an option for young women with contraindications to estrogen. Contraception is recommended in sexually active patients due to teratogenicity.

**Aromatase inhibitors (AI):** Over expression of aromatase has been identified in endometriotic implants (39). AI has been studied as part of combination treatment (with a progestin, CHC, or Danazol) to induce ovarian suppression (21,22). Side effects include vaginal dryness, hot flushes, and decreased BMD (20). Long term studies are needed. ESHRE suggests that AI should only be considered after hormonal treatment failure (20).

**Surgery:** Surgery should be considered after treatment failure extending to 3-6 months. For an adolescent, missing school/activities beyond this time can be particularly detrimental (43).

As the appearance of endometriotic lesions differs significantly in adolescents compared to adults, the operating gynaecologist should be familiar with diagnosis and treatment of endometriosis in this population (7,24,43). There is currently no evidence in adolescents suggesting that surgical treatment halts disease progression or prevents infertility (8). The American College of Obstetricians and Gynecologists recommends consideration of LNG-IUS placement at the time of laparoscopy for any patient with dysmenorrhea, chronic pain, or both (23).

**Post-operative considerations:** Patients should be counseled to continue hormonal treatment, as menstrual suppression reduces dysmenorrhea and endometrioma recurrence (7,20). There is no benefit to short post-operative courses of hormone treatment on pain, recurrence and fertility, thus ongoing medical treatment post-surgery is recommended unless fertility is imminently desired (19,24,50). An RCT in adults demonstrated a cure rate of 50% with surgery and 60% with combination of surgery and GnRH treatment, with reduced recurrence in combined treatment (65). Combination treatment demonstrated stability of endometriosis in 70% of adolescents after a mean interval of 29 months (1). A Cochrane Review demonstrated improved dysmenorrhea with post-operative medical treatment, however there was no effect on preventing pain recurrence when compared with surgery alone (66). Another review recommended post-operative long-term treatment with an emphasis on extended cycle, rather than cyclic use, of CHC to prevent recurrent retrograde menstruation and ovulation (50). Adolescents should be counselled on the possibility of recurrence as 30-50% of young women require repeat surgery within five years (19). Repeat surgery should be reserved for adolescents with pain more than two years post initial surgery despite ongoing medical treatment (7).

**Complementary medicine:** Women should be encouraged to disclose the use of complementary medicine to ensure there are no interactions with concurrent medications. Most of the adult studies described involve small sample sizes (67). Studies have examined Vitamins B1, E, and D, Omega 3 fatty acids, magnesium and ginger with modest or no effect (11,67). A recent Cochrane review demonstrated limited effectiveness for fenugreek, fish oil, fish oil plus vitamin B1, ginger, valerian, Vitamin B1, sataria, and zinc sulfate (31). A small RCT demonstrated improvement in sleep quality, daily pain, dysmenorrhea, dyspareunia, dyschezia and dysuria with the use of melatonin (68). A Cochrane review demonstrated improvement in dysmenorrhea, reduction in associated symptoms and reduced use of additional medications with the use of traditional Chinese medicine compared to placebo (27). A small randomized-controlled sham study of Japanese acupuncture in adolescents demonstrated initial improvement in pelvic pain at four weeks, although this difference waned at six months (69). Some patients with chronic pain may find an anti-inflammatory diet improves their daily pain, provided adequate nutrient intake is achieved.

**Support:** Adolescents with endometriosis experience significant effects on school, work and relationships (20). Young women with chronic pain should be screened for mental health illness and offered support (38). Collaborative care encompassing pain management, behavior modification, menstrual suppression and emotional support should be encouraged (9,38). More research is needed on psychological treatment for adolescents with CPP.

## Surveillance

Endometriosis induces a pro-inflammatory state affecting both pelvic anatomy and oocyte implantation (36,39), and infertility can be experienced in 30-50% (19). Young women should be counselled about future reproductive function. Endometriosis worsens with ongoing menstruation, and patients should be counselled on menstrual suppression until pregnancy is desired (19,24). Fertility rates are improved in women treated with hormone treatment and/or surgery (65). Endometriosis Is not associated with overall increased cancer risk, but there is an increased association with ovarian cancer, specifically endometrioid and clear cell histology types (9,20,36). It should be emphasized to the adolescent and her caregivers that the overall incidence of ovarian cancer is low.

## Genital Outflow Tract Obstruction

Recurrent cyclic abdominal pain in the absence of menses, or pain with first menses, should alert HCP to the possibility of abnormalities in the hymen or Müllerian structures (11). High suspicion is warranted in an adolescent with secondary sexual characteristics and amenorrhea two years beyond thelarche. Patients who are suspected to have an obstructive anomaly should be referred to a provider with expertise in this area, such as a pediatric gynaecologist. The patient can safely be placed on hormonal suppression until a qualified provider is available.

**Figure 1:**
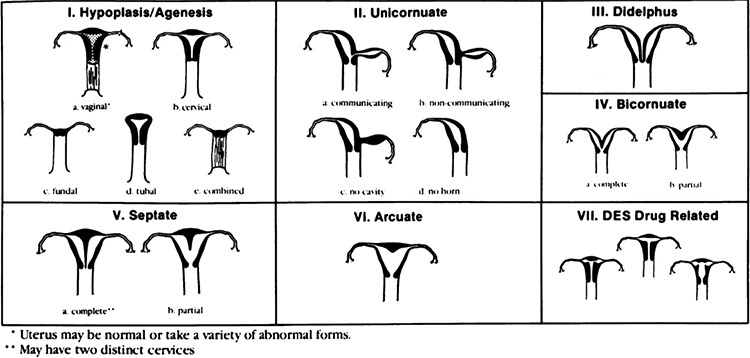
American Society for Reproductive Medicine Classification for Müllerian Anomalies

American Society for Reproductive Medicine Classification for Müllerian anomalies is seen in Figure 1 (70).

## Chronic Pelvic Pain

CPP is defined as pain lasting beyond three to six months that interferes with daily function (71). Prevalence in adult women is estimated at 14-16% (3). The differential diagnosis is similar to acute abdominal pain (Table 1). Gynecologic etiologies should be considered in post- and peri-pubertal adolescents, including endometriosis, adenomyosis, pelvic inflammatory disease, ovarian cysts, pelvic venous congestion and pelvic adhesions (3). Central sensitization, the severe experience of pain that results from multiple lower-level pain stimuli over time, can develop due to longstanding dysmenorrhea (16). Symptoms of sensitization include daily pain, nausea, dizziness, anxiety, depression, insomnia, and skin sensitivity. In CPP patients, whereby hormonal treatment or gynecological cause are absent, a musculoskeletal etiology of chronic pain was demonstrated in 67% (72). Chronic abdominal pain can precipitate hypertonicity/spasm in the abdominal wall. Attention should also be given to aggravating factors including biomechanical stressors (poor posture, footwear, heavy bags) and acute muscle strain (10). Mobilization of multi-disciplinary teams is important to address comorbidities (17). Treatment includes assessment and improvement in biomechanical stressors, heat, rest, NSAIDs, and trigger point injections (10). The adolescent may require referral to physiotherapist specially trained in pelvic floor physiotherapy.

## Support

CPP can affect an adolescent at school, at home, or with peers. It is important to validate the pain and its impact. Patients should be educated on the pathophysiology of pain, and the role of pain sensitization in symptom experience (12,17). Pain catastrophizing is prominent in CPP patients and is associated with higher pain levels and impaired quality of life. Screening for mental health illness should be performed, as this can contribute to or worsen CPP. Adolescents may benefit from pharmacologic, cognitive and behavioral treatment and mindfulness training. The adolescent and involved caregivers should be involved in the generation of a treatment plan, including management of pain crisis (10,17). Treatment of mental health disorders and reduction in stress will reduce pain (12). The use of opioid narcotics should be discouraged (23). Neuropathic medications (amitriptyline, serotonin-noradrenaline re-uptake inhibitors, anticonvulsants) may be trialed (17). The adolescent should be supported in continuing with education and extra-curricular activities. Physical activity is important for overall health, and is viewed as the “best non-drug treatment for pain” (17).

## Summary

The majority of adolescents will experience discomfort during menstruation. HCP should avoid normalization of dysmenorrhea, as young women are missing out on educational, social and vocational opportunities. The safety and effectiveness of NSAIDs, CHCs, progestin only options, and GnRHa have been outlined. HCP should not delay this treatment to complete physical examination and/or investigations. Patients with persistent pain despite medical treatment should be further investigated, and a diagnosis of endometriosis should be considered and a treatment plan developed. Multi-disciplinary teams should address biopsychosocial contributors to pain. CPP in the adolescent population requires further research into whether outcomes seen in the adult population can be translated and/or modified for youth. By reducing barriers to treatment and increasing the focus on high quality research in the adolescent population, we can improve the overall health outcomes for young women.

## Practice Points

1. It is safe to offer menstrual suppression with combined hormonal contraception and progestin-only options to adolescents with dysmenorrhea.

2. Surgery with the aims at of diagnosis and treatment should be considered when medical treatments do not provide relief.

3. Adolescents with suspected or confirmed endometriosis should be recommended for menstrual suppression until fertility is desired.

4. In adolescents, add-back treatment should be offered concurrently with the initiation of GnRHa.

5. Young women with CPP should be followed by a multi-disciplinary team.

## Figures and Tables

**Table 1:**
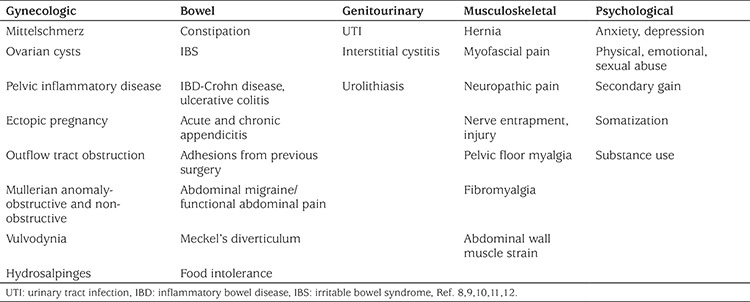
Differential diagnosis for pelvic pain

**Table 2:**
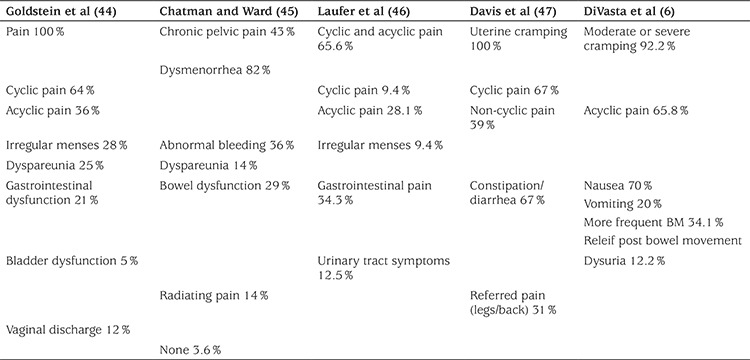
Symptoms in adolescents with endometriosis

**Table 3:**
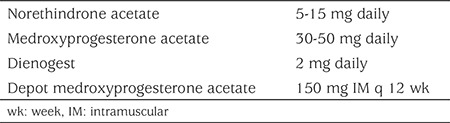
Dosage regimens for progestin only options
